# Mental Health Problems and Internet Access: Results From an Australian National Household Survey

**DOI:** 10.2196/14825

**Published:** 2020-05-15

**Authors:** Lay San Too, Liana Leach, Peter Butterworth

**Affiliations:** 1 Centre for Mental Health Melbourne School of Population and Global Health The University of Melbourne Parkville Australia; 2 National Centre for Epidemiology and Population Health Research School of Population Health The Australia National University Canberra Australia; 3 Melbourne Institute of Applied Economic and Social Research The University of Melbourne Parkville Australia; 4 Centre for Research on Ageing, Health & Wellbeing Research School of Population Health The Australia National University Canberra Australia

**Keywords:** internet access, mental health, affordability, mobile phone

## Abstract

**Background:**

Mental health support and interventions are increasingly delivered on the web, and stepped care systems of mental health services are embracing the notion of a digital gateway through which individuals can have access to information, assessment, and services and can be connected with more intensive services if needed. Although concerns have been raised over whether people with mental health problems are disadvantaged in terms of their access to the internet, there is a lack of representative data on this topic.

**Objective:**

This study aimed to examine the relationship between mental health and internet access, particularly lack of access because of affordability issues.

**Methods:**

Data from wave 14 of the *Household, Income, and Labour Dynamics in Australia* survey were used (n=15,596) in the analyses. Sample weights available in the survey were used to calculate the proportion of those with or without internet access for those with and without mental health problems and more severe long-term mental health conditions. These proportions were also calculated for those with and without internet access due, specifically, to affordability issues. Multinomial logistic regression analyses assessed the relationship between mental health status and internet access/affordability issues, adjusting for a range of covariates.

**Results:**

Access to the internet was poorer for those with mental health problems (87.8%) than those without mental health problems (92.2%), and the difference was greater when a measure of more severe mental health conditions was used (81.3% vs 92.2%). The regression models showed that even after adjusting for a broad range of covariates, people with mental ill health were significantly more likely to have no internet access because of unaffordability than those without mental ill health (mental health problems: relative risk ratio [RRR] 1.68; 95% CI 1.11-2.53 and severe mental health conditions: RRR 1.92; 95% CI 1.16-3.19).

**Conclusions:**

As Australia and other nations increasingly deliver mental health services on the web, issues of equity and affordability need to be considered to ensure that those who most need support and assistance are not further disadvantaged.

## Introduction

### Background

In Australia, as in many other countries, access to and use of the internet has become commonplace [1-3]. In 2016-2017, a regular national survey conducted by the Australian Bureau of Statistics (ABS) found that 86.5% of Australians aged 15 years or older had accessed the internet for personal use in the previous 3 months [2], with their most frequent web-based activities being entertainment (80.0%), social networking (79.9%), banking (79.5%), and shopping (72.6%). In addition, 46.1% had accessed web-based health-related services. The use of the internet (or broader digital platforms) for the delivery of mental health services, information, and support (electronic mental health) is seen as a mechanism to address the barriers associated with traditional mental health services (eg, cost and accessibility).

A large number of web-based interventions have been developed and delivered for treating mental illness. There is evidence supporting the efficacy of some of these interventions [4-8]. For example, a recent meta-analysis included 13 studies comparing internet-delivered cognitive behavioral therapy (ICBT) and face-to-face cognitive behavioral therapy (CBT), and the results showed that ICBT was as equally effective as face-to-face CBT in treating depression and anxiety disorders [6]. A meta-analysis of 12 randomized controlled trials comparing ICBT and control groups (eg, treatment as usual, waiting list, and attention placebo) showed that ICBT had a large effect in treating anxiety disorders and a small effect in treating depression [7]. This study also showed that ICBT with minimal therapist support had a large effect size and ICBT without such support had a small effect size. Apart from ICBT, web-based psychoeducational programs and self-management interventions have also been shown to be effective in improving psychiatric symptoms [5]. However, it must also be acknowledged that some research have raised concerns that although various web-based interventions appear to be effective in research trials, their real-world efficacy may be limited by low adherence [9], the severity of patients’ illness, lack of support, and inadequate personalization of program content [10,11].

In response to a recent review of mental health programs and services [12], the Australian government announced the introduction of a stepped care approach to service delivery, encompassing a hierarchy of services of increasing intensity [13]. A digital gateway is the entry point for services, providing a greater focus on early intervention. This digital mental health gateway, *Head to Health*, offers information about mental health services and resources delivered by mental health service providers on the web, including free/low-cost apps (both self-help and clinician-moderated options), online support communities, web-based courses, and phone services. Although *Head to Health* may assist many Australians seeking mental health care (particularly those who prefer anonymity or who live far from face-to-face services), it cannot be considered a universal platform, when a digital divide remains in Australia. Engagement by some sociodemographic groups will be lower because the accessibility and use of the internet remain, to some extent, socioeconomically determined [14]. Those with low levels of educational attainment, who are unemployed, in low-income households, and/or live in rural or remote areas are less likely to have access to the internet and are more likely to have low internet literacy [2,15-18]. According to the ABS, although the proportion of households with access to the internet at home has steadily increased since 2004-2005, it has plateaued at 86% between 2014-2015 and 2016-2017 [2]. The ABS survey did not explicitly ask why people did not use the internet, but their report shows that affordability is likely a major factor, given that internet use was much less prevalent among households in the lowest quintile of household income (68.8%) compared with those in the high-income quintile (97.4%). Unfortunately, people with less education and fewer financial resources are also particularly vulnerable to mental health problems [19,20], and thus, those most in need of low-cost web-based mental health interventions are likely to be those least likely to be able to access this resource.

Several studies have examined internet use by people experiencing mental illness. In a recent study, Robotham et al [21] surveyed 241 people with psychosis or depression in London about their use of the internet and internet-enabled technologies. They found that only 10% of their sample was digitally excluded, with limited internet access. For people with psychosis, the most commonly reported barriers to using the internet were security concerns, lack of credit/money, lack of knowledge, lack of places to access the internet, and lack of availability. For people with depression, the most common barriers were security concerns and a lack of credit/money. They also found that for people with psychosis, those digitally excluded tended to be older and have been in contact with mental health services for a longer period. In contrast, Tobitt and Percival [22] interviewed 97 users of community mental health rehabilitation services in London and found high levels of digital exclusion. Only 14.4% of their sample used the internet, 17.5% used computers, and 40.2% used mobile phones. Those who used these technologies were younger than those who did not. Those who used mobiles and computers were more likely to reside in low-support/high-independence placements. Although these recent studies suggest there may be accessibility issues for people experiencing mental illness, their conclusions are limited by small sample sizes and no comparison with people without mental illness. Overall, although evidence about accessibility/engagement is critical to the success of web-based interventions for people experiencing mental illness, knowledge remains scarce. Thus, additional research on this issue is urgently needed.

### Objective

This study sought to address the lack of population-based research examining the relationship between mental health and internet access. We used data from a large, nationally representative Australian survey. We hypothesized that people with mental health problems would be more likely to have no access to the internet compared with those without mental health problems, mainly because of affordability issues. We also hypothesized that this relationship would be more evident for those who had a severe mental health condition.

## Methods

### Participants

This study used data from the *Household, Income, and Labour Dynamics in Australia* (HILDA) survey. HILDA is a longitudinal study of a nationally representative sample of Australian households, randomly selected through a multistage approach [23]. It has been conducted annually since 2001 and collects household and individual information across a wide range of domains (eg, demographic, social, health, and financial) through face-to-face interviews and self-completion questionnaires. Information at the individual level is collected from all household members aged 15 years or older in each survey wave.

In wave 1, 13,969 individuals from 7682 households responded to the survey (66% response rate) [24]. Retention of these individuals in wave 2 was 87% and more than 90% for each wave thereafter. The total number of respondents in subsequent waves was greater than those in wave 1 for several reasons, including (1) nonrespondents from participating households in wave 1 were successfully interviewed in later waves, (2) individuals in sample households turned 15 years old, and (3) individuals were added to the sample following changes in household composition (eg, marriage of a household member). In addition, to retain the cross-sectional representativeness of the HILDA sample, the sample was topped up by including an additional 3652 people from 2153 households in wave 11. Each wave of the HILDA survey comprises a household survey with a key informant from each household and a personal interview and a (paper) self-complete questionnaire with all household members aged 15 years or older. The self-complete questionnaire is either completed during the interview process or left with respondents to complete at a later time. As a result, the response rate to the self-complete questionnaire is lower than that of the interview, with an average return rate of 90%.

This study primarily used data from wave 14 (collected in 2014) when a measure of material deprivation was included. This module of questions examined which resources, activities, and services were considered essential and if each household had access to them (total number of responding households at wave 14=8327, 87.3% of all invited households). The measure of mental health problems was drawn from the self-complete questionnaire, meaning there was a maximum of 15,596 respondents (75.8% of all invited persons) included in the analyses.

### Ethics Approval

The HILDA survey was approved by the Human Research Ethics Committee of the University of Melbourne.

### Measures

#### Outcome Variable

The key household respondent answered the material deprivation module on behalf of the household. The module considered 26 different resources, activities, and services (eg, *access to the internet at home*, *a decent and secure home*, and *medical treatment when needed*) [23] and asked respondents (1) if they considered this essential (response options: *yes* and *no*); (2) if the household had this item (response options: *yes* and *no*); and (3) if not, whether this was because they could not afford it (response options: *yes* and *no*). On the basis of the latter 2 questions, we created a variable regarding internet access: (1) yes, the household had internet; (2) no, the household did not have internet because of unaffordability; and (3) no, household did not have internet for reasons other than unaffordability.

#### Exposure Variables

Each household member aged 15 years or older was invited to complete a personal interview and the self-completion questionnaire, which included mental health questions. Mental health was assessed using the 5-item Mental Health Inventory (MHI-5), a subscale of the Medical Outcomes Study 36-item short-form health survey. This survey is administered annually in HILDA (ie, at every wave) and is one of the most widely used self-completion measures of health status [25,26]. The MHI-5 has been widely recognized as an effective screening instrument for depression/common mental disorders in both the general population and clinical settings [27,28]. The MHI-5 score (ranging from 0 to 100) was dichotomized at an established cutoff point, with below 50 indicating mental health problems [29].

Mental health was also assessed using mental health items from a module of questions assessing the presence of any long-term health condition that restricted activity. This mental health measure was based on 2 questions assessing *any mental illness that requires help or supervision* and a *nervous or emotional condition that requires treatment*. The need for help, supervision or treatment indicated greater adverse functional effects and thus a more severe form of mental health condition. Those who responded *yes* to either of these questions were classified as having a severe mental health condition, whereas everyone else was classified as not having a severe mental health condition.

### Covariates

Variables that are known to be associated with mental health and that might potentially confound the relationship between mental health and internet access were adjusted in the analysis [21,22]. These include sex, age, partner status, any children under 15 years old in the household, employment status, annual household gross income, financial hardship, and residential remoteness. In terms of partner status, those who were legally married or in a de facto relationship were grouped as *having a partner*, whereas those who were separated, divorced, widowed, or never married/not in a de facto relationship were grouped as *not having a partner*. Employment status included the categories of employed, unemployed, and not participating in the labor force. Annual household gross income was the sum across all household members of financial year market income, private transfers, Australian and foreign pensions and benefits, and irregular income (described in detail elsewhere [30]). Household income was classified into 5 groups. Financial hardship was based on 7 items: (1) *could not pay electricity, gas, or telephone bills on time*; (2) *could not pay the mortgage or rent on time*; (3) *pawned or sold something*; (4) *went without meals*; (5) *was unable to heat home*; (6) *asked for financial help from friends or family*; and (7) *asked for help from welfare/community organizations*. Respondents were classified as experiencing financial hardship if they responded *yes* to any of these items. Residential remoteness was classified into 5 categories based on the 2011 Australian Statistical Geography Standard: major cities, inner regional, outer regional, remote, and very remote.

### Statistical Analysis

All analyses applied the sample weights (ie, household population weight for items from the household survey and responding person sample weight for items from the self-complete questionnaire, details of the weighting methodology described elsewhere [31]) provided in the HILDA dataset to reflect the overall Australian population. We used descriptive statistics to report the sample characteristics and the proportion of those with or without internet access for those with and without mental health problems and more severe long-term mental health conditions. For each weighted percentage, we reported the unweighted number which may differ from the weighted estimate. Multinomial logistic regression models were then used to assess the relationship between mental health status (assessed separately by MHI-5 cutoff point and questions on any long-term health condition) and internet access. The first model was a simple bivariate model, and the subsequent models adjusted for age and sex (model 2), partner status and any children aged under 15 years (model 3), residential remoteness (model 4), and socioeconomic circumstances (employment status, household income, and financial hardship; model 5). Finally, we tested the interaction effect of mental health with age by comparing a model that comprised only the main effects with a model that also incorporated their interaction effect using the likelihood ratio test.

The proportion of observations with missing data on all variables was low, ranging from 0% to 3.2% (financial hardship). Our analyses were based on observations with no missing data (complete analyses). All analyses were performed using the Stata/SE version 14 (StataCorp LLC).

### Sensitivity Analyses

We performed a sensitivity analysis using HILDA data from the following year (wave 15, collected in 2015). This wave did not include the deprivation module and did not assess if respondents considered internet access to be essential but asked each participant if they had access to the internet at home (response categories: *yes* or *no*). Wave 15 also included the Kessler Psychological Distress Scale (K10) [32], a widely used and validated scale used to assess nonspecific psychological distress and screen for mental disorders in the population. The validity of K10 as an indicator of distress is comparable with that of the MHI-5 [29]. For this analysis, we used recommended cutoff points to identify those with very high psychological distress as an indicator of mental health problems [33]. Sample weights were used to estimate the proportion of those with or without internet access for those with and without very high psychological distress and in logistic regression models to examine the relationship between mental health and internet access, controlling for the same covariates as the main analyses.

## Results

### Sample Characteristics

Of the respondents in wave 14, there were approximately equal-weighted proportions of males (49.2%) and females (50.8%) as well as respondents who were aged less than 35 years (34.8%), between 35 and 54 years (33.5%), and more than 54 years (31.7%; Table 1). Approximately 60% of respondents had a partner, 31.0% had children under 15 years old, 3.9% were unemployed, about half lived in a household with an annual gross income of more than Aus $100,000 (US $63,868), 21.7% experienced financial hardship, and 71.4% resided in major cities.

The cutoff point of 50 on the MHI-5 defined approximately 11% of HILDA survey respondents with mental health problems. For comparison, in the analysis of the 2014-2015 National Health Survey, the ABS identified 11.7% of Australian adults with high or very high levels of psychological distress assessed using K10, a measure that is comparable with MHI-5 [34]. Approximately 5% of the HILDA survey respondents were identified with long-term mental health conditions that required help/supervision or treatment.

Just less than half of the households (46%, as represented by the key respondent) indicated that internet access at home was essential. This was somewhat greater than the percentage of households where it was considered essential to have a television (44.1%) but less than a motor vehicle (55.1%). It is estimated that 88.5% of Australian households had access to the internet (consistent with the ABS estimate of 86.1%). At the person level, approximately 91.6% of respondents resided in households that had access to the internet, whereas 1.5% were in households with no internet access because of not being able to afford it, and 6.8% were in households with no internet access due to other reasons.

**Table 1:** Weighted proportion of sample characteristics at wave 14 (persons: n=15,596 and households: n=8327).

Characteristics		Weighted percentage	
		n (%)	95% CI
**Sex**			
	Male	7308 (49.21)	48.14-50.28
	Female	8288 (50.79)	49.72-51.86
**Age group (years)**			
	<35	5355 (34.78)	33.72-35.86
	35-54	5037 (33.48)	32.47-34.50
	>54	5204 (31.74)	30.82-32.67
**Partner status**			
	No partner	5734 (39.41)	38.36-40.47
	Have a partner	9861 (60.59)	59.53-61.64
**Children <15 years old**			
	No	10,939 (69.05)	68.05-70.03
	Yes	4657 (30.95)	29.97-31.95
**Employment status**			
	Employed	9771 (62.13)	61.09-63.17
	Unemployed	634 (3.94)	3.46-4.47
	Not in the labor force	5191 (33.93)	32.94-34.93
**Annual household gross income Aus $ (US $)**			
	<34,000 (21,715)	1978 (10.33)	9.83-10.86
	34,000-59,999 (21,715-38,320)	2509 (15.41)	14.68-16.18
	60,000-99,999 (38,321-63,867)	3361 (21.43)	20.57-22.31
	100,000-159,999 (63,868-102,187)	3995 (26.48)	25.61-27.38
	>160,000 (102,188)	3753 (26.34)	25.46-27.25
**Financial hardship**			
	No	11,723 (78.34)	77.44-79.22
	Yes	3372 (21.66)	20.78-22.56
**Remoteness**			
	Major cities	10,452 (71.43)	70.59-72.26
	Inner regional	3374 (18.85)	18.16-19.55
	Outer regional	1570 (8.48)	8.00-8.99
	Remote/very remote	200 (1.24)	1.03-1.49
**Mental health problems (5-item Mental Health Inventory)**			
	No	13,888 (89.19)	88.53-89.81
	Yes	1662 (10.81)	10.19-11.47
**Severe mental health conditions**			
	No	14,768 (94.99)	94.56-95.39
	Yes	825 (5.01)	4.61-5.44
**Internet access is essential (household respondent)**			
	No	4677 (53.98)	52.58-55.38
	Yes	3617 (46.02)	44.62-47.42
**Households with internet access**			
	No	1020 (11.55)	10.73-12.42
	Yes	7301 (88.45)	87.58-89.27
**Persons with internet access at home**			
	Yes	14,185 (91.62)	91.08-92.13
	No, cannot afford	259 (1.53)	1.26-1.86
	No, other reasons	1143 (6.85)	6.42-7.31

### Weighted Proportion of Internet Access by Mental Health Variables

Table 2 shows that similar weighted proportions of respondents with and without mental health problems (MHI-5) were from households in which having internet access at home was considered essential (44.9% vs 46.2%). However, a lower weighted proportion of respondents with mental health problems had access to the internet compared with those without mental health problems (87.8% vs 92.2%). In addition, the weighted proportion of respondents with mental health problems who did not have internet access because they could not afford it was more than three times greater than it was for respondents without mental health problems (4.0% vs 1.3%). The difference in the weighted proportion of those with and without mental health problems who had no internet access at home because of other reasons was minor, 8.3% and 6.5%, respectively.

**Table 2:** Weighted proportion of internet access by mental health problems (5-item Mental Health Inventory) and severe mental health conditions.

Internet access		Mental health problems (5-item Mental Health Inventory)				Severe mental health conditions			
		No		Yes		No		Yes	
		n (%)	95% CI	n (%)	95% CI	n (%)	95% CI	n (%)	95% CI
**Internet access is essential** **(household respondent)**									
	No	4153 (53.82)	52.32-55.31	508 (55.10)	51.06-59.07	4425 (53.84)	52.39-55.29	250 (56.56)	51.26-61.71
	Yes	3242 (46.18)	44.69-47.68	367 (44.90)	40.93-48.94	3421 (46.16)	44.71-47.61	195 (43.44)	38.29-48.74
**Persons with internet access at home**									
	Yes	12,718 (92.17)	91.59-92.72	1437 (87.77)	85.66-89.60	13,504 (92.20)	91.64-92.73	678 (81.27)	77.38-84.62
	No, cannot afford	188 (1.29)	1.03-1.63	66 (3.97)	2.91-5.39	210 (1.41)	1.14-1.75	49 (5.17)	3.60-7.37
	No, other reasons	974 (6.53)	6.06-7.04	159 (8.27)	6.79-10.02	1045 (6.38)	5.94-6.86	98 (13.56)	10.58-17.23

A similar pattern was observed for the measure of severe mental health conditions, but the difference was greater in magnitude. Specifically, the difference in the weighted proportion of persons with internet access at home between those with and without severe mental health conditions was 10.9% (81.3% vs 92.2%). The weighted proportion of those with severe mental health conditions who had no internet because they could not afford it was 4 times greater than it was for those without this condition (5.2% vs 1.4%). The difference in the weighted proportion of those with and without mental health problems who had no internet access at home because of other reasons was 7.2% (6.4% vs 13.6%).

### Relationship Between Mental Health and Internet Access

A series of models examining the relationship between mental health and internet access at home are presented in the Multimedia Appendix 1. The simple model shows that the relative risk that people with mental health problems did not have access to the internet at home because they could not afford it was 3 times greater than for those without mental health problems (relative risk ratio [RRR] 3.22; 95% CI 2.16-4.80). People with mental health problems were also more likely to have no access to the internet at home for *other reasons* than those without mental health problems, although the relative risk was lower (RRR 1.33; 95% CI 1.06-1.67). These results remained significant in the subsequent models adjusted for sex, age, partner status, young children, and remoteness (no internet access because of unaffordability; RRR 2.60; 95% CI 1.73-3.93 and no internet access because of *other reasons*: RRR 1.39; 95% CI 1.08-1.80). The greater risk of those with mental health problems having no internet access because of unaffordability was attenuated but remained significant after adjusting for the comprehensive block of financial measures (employment status, household income, and financial hardship; RRR 1.68; 95% CI 1.11-2.53), but the difference associated with mental health in having no access to the internet for *other reasons* was no longer significant in this final model.

In the final model (Multimedia Appendix 1), other covariates that were related to no internet access because of unaffordability included did not have a partner, had children aged under 15 years, from a low-income household, experienced financial hardship, and lived in the outer regional area. Those aged older than 34 years, who did not have a partner, did not have children aged under 15 years, were not in the labor force, from a low-income household, and lived in regional/remote areas were more likely to report no internet access for *other reasons*.

As shown in Multimedia Appendix 2, individuals with a severe mental health condition were more likely to report no internet access because they could not afford it (model adjusted for sociodemographic variables and remoteness: RRR 3.46; 95% CI 2.20-5.45 and fully adjusted model: RRR 1.92; 95% CI 1.16-3.19) compared with those without a severe mental health condition. Those with a severe mental health condition were also more likely to report no internet access because of *other reasons* (model adjusted for sociodemographic variables and remoteness: RRR 2.06; 95% CI 1.46.2.91 and fully adjusted model: RRR 1.43; 95% CI 1.01-2.01). The covariates that were significant in the final model (Multimedia Appendix 2) were the same as those in Multimedia Appendix 1.

The likelihood ratio test showed that there was no significant interaction between mental health (assessed by MHI-5 and questions on any long-term health condition) and age, indicating no significant difference in the relationship between mental health and internet access at home across age groups.

### Findings From Sensitivity Analyses

When using wave 15 data, we found 91.3% (consistent with 91.7% in 2014) of respondents reported that they had access to the internet, which included 87.4% of those with very high psychological distress (based on the K10) and 91.9% without very high distress (Multimedia Appendix 3). The weighted logistic regression models confirmed that those with very high distress were significantly more likely to report having no internet access at home than those without distress (model adjusted for sociodemographic variables and remoteness: odds ratio [OR] 2.14, 95% CI 1.61-2.84 and full model incorporating socioeconomic measures: OR 1.46, 95% CI 1.08-1.97; Multimedia Appendix 4).

## Discussion

### Principal Findings

This study aimed to examine the relationship between mental health and internet access using a large representative Australian sample. The findings show that in 2014, 91.7% of respondents had internet access at home. However, the proportion was lower among those identified with mental health problems (87.8%) compared with those without (92.2%) mental health problems. This difference was greater for those with than those without severe mental health conditions (81.3% vs 92.2%). The findings also show that individuals with mental health problems were less likely to have internet access at home because they could not afford it compared with those without mental health problems: an effect that remained even after controlling for an extensive range of sociodemographic, geographical, and socioeconomic factors. After controlling for these factors, mental health problems were no longer associated with not having internet access because of reasons other than unaffordability. The same pattern of results on not having internet access because of affordability issues (with a greater RRR) was observed when using severe mental health conditions as the exposure variable. However, severe mental health conditions remained significantly associated with lack of internet access because of reasons other than unaffordability in the fully adjusted model.

### Comparison With Prior Work

Our study analyzed data from the HILDA survey, a large, high-quality, nationally representative household panel survey. The weighted estimate derived from the data of the proportion of Australian households with internet access (88.5%) was consistent with the published figures from the ABS for the same years (2014-2015, 85.9%) [2], further enhancing our confidence in the robustness of the findings. The measure of mental health problems used in the main models, based on the MHI-5, assesses symptoms associated with common mental disorders such as depression and generalized anxiety. Using this measure, we found a difference of 4.4% in internet access at home between those with and those without mental health problems in 2014. Other analyses included the alternative marker of mental health conditions that identified those who reported having a long-term mental illness that required help or supervision or required treatment. We hypothesize that this measure may identify a cohort with more severe mental health conditions. Accordingly, we found a much greater difference (10.9%) in the rate of internet access at home among those with and without such a mental health condition and evidence of a much stronger association between this form of mental health condition and lack of internet access at home for financial reasons (RRR 1.92 vs MHI-5’s RRR 1.68). This provides evidence of an inverse relationship between the severity of mental health conditions and likelihood of internet access and further strengthens the importance of understanding the nature of, and how to potentially address, this digital exclusion of those with poor mental health before the design and implementation of digital-centric mental health service systems.

Consistent with our hypothesis, we found that individuals with mental health problems are more likely to not have access to the internet because of affordability issues. This finding echoes previous research with nonrepresentative samples that have reported inadequate financial resources as a barrier to internet use for people with mental illness [21]. The same issue has also been emphasized in the 2017 Australian Digital Inclusion Index [35], where affordability remained a key challenge for some excluded groups. In addition, we found that not having internet access because of reasons other than unaffordability was not related to mental health measured by the MHI-5 but was marginally related to more severe mental health conditions in the fully adjusted models. This finding indicates the likelihood that people with more severe mental health conditions have no internet access primarily because of unaffordability and secondarily because of other potential reasons, such as lack of internet literacy, low acceptability of internet use, and concerns related to cybersecurity and privacy [21,36].

The findings of this study have important implications in terms of ensuring equity in the planning, delivery, and provision of web-based mental health interventions and their role in broader mental service systems. Although digital platforms provide a low-cost option for the delivery of effective mental health services and treatments and can have a key role as a central gateway directing individuals with mental health problems to appropriate services, our findings suggest that the key target for these services (potentially both those likely to benefit from web-based self-help and more intensive assistance) will be disproportionally less able to access these treatments from home. As affordability is a key factor for lack of internet access in people with mental illness, making internet access more affordable to this vulnerable group of people may be one way to address these inequalities. This study raises questions about the use of web-based mental health service delivery as the gateway to services and support. It points to the need for alternative and flexible pathways to care, and that it is essential to continually monitor service accessibility and engagement with the most disadvantaged in our community. Although the evolution of web-based service delivery will continue, it is critical to note that the digital divide remains.

The ongoing inequality in internet access documented in this paper also has implications for those who undertake mental health research via web-based platforms or who develop and evaluate web-based mental health interventions. The results of this study suggest that, if not explicitly targeted, those with mental health problems (and more so, those with more severe mental conditions) will be underrepresented in this research and that caution is needed in the interpretation and generalizability of the findings.

### Strengths and Limitations

The key strengths of this study include the large nationally representative sample and the multiple and robust assessments of mental health problems. However, there are some limitations that should be considered in the interpretation of the findings. First, although our analysis produced estimates of household internet access consistent with published figures from the ABS, as in all longitudinal surveys, the HILDA sample may be influenced by selection and attrition bias, which, over time, may limit the generalizability of the findings. To overcome such potential bias, cross-sectional and longitudinal weights are generated for each wave in the HILDA. The application of these weights to adjust for differences between the characteristics of the HILDA sample and the characteristics of the Australian population corrects for the under- or over-representativeness in the sample of certain characteristics. The application of the population weights included in the HILDA allows us to make stronger inferences about the Australian population from the HILDA survey data. The inclusion of new household members and the addition of a top-up sample in 2011 also increased the representativeness of the HILDA sample (eg, providing an opportunity to recruit newly arrived Australian residents). Second, the HILDA study only includes self-report data on mental health and internet access, and thus, the observed relationship may be understated or overstated because of under- or over-reporting resulting from recall bias. Nonetheless, the consistency of findings from the 2014 (in which a single key household respondent reported on household internet access) and the 2015 HILDA survey (where each individual respondent provided data on household internet access and their own mental health using a different measure) may suggest such effects were not substantial. Third, the HILDA survey only asks about internet access at home in a general manner and, therefore, could be interpreted by respondents narrowly to imply that only fixed-line access was being accessed. However, the correspondence of the HILDA survey estimates of household internet access with the estimate from the ABS, in which connection to the internet at home via a mobile phone or smartphone is explicitly described, suggests that the approach in the HILDA survey did not significantly underestimate the levels of household internet access. Fourth, we have no detailed information about the other reasons people did not have internet access at home (other than unaffordability), so we do not know if this reflects a personal choice, broader geographic access difficulties, or other reasons. Finally, the HILDA survey does not contain questions about internet use related to accessing web-based mental health services. Thus, although we assume that those with internet access have less opportunity to engage with web-based services, this topic could be included in future questionnaires.

### Conclusions

In summary, the findings provide direct evidence that people with mental health problems are more likely to not have access to the internet because of affordability issues than those without mental health problems (particularly those with more severe mental health conditions). This means people who need web-based medical treatments the most are less able to access them. Translating our findings to the context of Australia’s new digital mental health gateway, we suggest that there are inequity issues and that everyone with a mental health problem cannot currently access what is intended to be a universal platform. The problem of internet affordability needs to be addressed to increase the accessibility of web-based treatments for everyone.

## Appendix

Multimedia Appendix 1 Relative risk ratio and 95% CIs from multinomial logistic regression models assessing the relationship between mental health problems (5-item Mental Health Inventory) and internet access at home.

Multimedia Appendix 2 Relative risk ratio and 95% CIs from multinomial logistic regression models assessing the relationship between severe long-term mental health conditions and internet access at home.

Multimedia Appendix 3 Weighted proportion of internet access by mental health condition in wave 15.

Multimedia Appendix 4 Odds ratio and 95% confidence intervals from logistic regression models assessing the relationship between psychological distress and no internet access at home (wave 15).

## Abbreviations

ABS: Australian Bureau of Statistics
CBT: cognitive behavioral therapy
DSS: Department of Social Services
HILDA: Household, Income, and Labour Dynamics in Australia
ICBT: internet-delivered cognitive behavioral therapy
K10: Kessler Psychological Distress Scale
MHI-5: 5-item Mental Health Inventory
OR: odds ratio
RRR: relative risk ratio
