# Supplementary material for: Mental Health Problems and Internet Access: Results From an Australian National Household Survey
Source: JMIR Ment Health. 2020 May 15;7(5):e14825. doi: 10.2196/14825 (PMC7260658; doi:10.2196/14825)
Supplement: Multimedia Appendix 1 [file mental_v7i5e14825_app1.docx]

| Base outcome (internet access: yes) | | | Model 1, RRR^a^ (95% CI) | Model 2 (adding sex and age), RRR (95% CI) | Model 3 (adding partner status and children aged <15 years), RRR (95% CI) | Model 4 (adding remoteness), RRR (95% CI) | Model 5 (adding employment, household income, and financial hardship), RRR (95% CI) |
| --- | --- | --- | --- | --- | --- | --- | --- |
| **Relative outcome (internet access: no, cannot afford)** | | | | | | | |
|  | **Mental health problems** | | | | | | |
|  |  | No (reference) | 1.00 | 1.00 | 1.00 | 1.00 | 1.00 |
|  |  | Yes | 3.22 (2.16-4.80)^b^ | 3.27 (2.18-4.91)^b^ | 2.60 (1.73-3.91)^b^ | 2.60 (1.73-3.93)^b^ | 1.68 (1.11-2.53)^c^ |
|  | **Sex** | | | | | | |
|  |  | Male (reference) | N/A^d^ | 1.00 | 1.00 | 1.00 | 1.00 |
|  |  | Female | N/A | 1.26 (0.86-1.85) | 1.12 (0.74-1.70) | 1.13 (0.74-1.71) | 0.91 (0.57-1.48) |
|  | **Age group (years)** | | | | | | |
|  |  | <35 (reference) | N/A | 1.00 | 1.00 | 1.00 | 1.00 |
|  |  | 35-54 | N/A | 0.67 (0.41-1.09) | 1.26 (0.75-2.12) | 1.23 (0.73-2.07) | 0.88 (0.50-1.54) |
|  |  | >54 | N/A | 1.23 (0.77-1.97) | 2.68 (1.51-4.78)^e^ | 2.53 (1.42-4.49)^e^ | 1.71 (0.86-3.41) |
|  | **Partner status** | | | | | | |
|  |  | No partner (reference) | N/A | N/A | 1.00 | 1.00 | 1.00 |
|  |  | Have a partner | N/A | N/A | 0.15 (0.10-0.22)^b^ | 0.15 (0.10-0.21)^b^ | 0.22 (0.15-0.33)^b^ |
|  | **Children aged <15 years** | | | | | | |
|  |  | No (reference) | N/A | N/A | 1.00 | 1.00 | 1.00 |
|  |  | Yes | N/A | N/A | 1.82 (1.14-2.90)^c^ | 1.77 (1.11-2.81)^c^ | 2.02 (1.16-3.50)^c^ |
|  | **Employment status** | | | | | | |
|  |  | Employed (reference) | N/A | N/A | N/A | N/A | 1.00 |
|  |  | Unemployed | N/A | N/A | N/A | N/A | 1.93 (0.94-3.97) |
|  |  | Not in the labor force | N/A | N/A | N/A | N/A | 1.30 (0.77-2.18) |
|  | **Annual household gross income** | | | | | | |
|  |  | <AU $34,000 (US $ 21,715) (reference) | N/A | N/A | N/A | N/A | 1.00 |
|  |  | AU $34,000-AU $59,999 (US $21,715-US $38,320) | N/A | N/A | N/A | N/A | 0.99 (0.58-1.67) |
|  |  | AU $60,000-AU $99,999 (US $ 38,321-US $63,867) | N/A | N/A | N/A | N/A | 0.44 (0.26-0.75)^e^ |
|  |  | AU $100,000-AU $159,999 (US $63,868-US $ 102,187) | N/A | N/A | N/A | N/A | 0.06 (0.03-0.14)^b^ |
|  |  | >AU $160,000 (US $102,188) | N/A | N/A | N/A | N/A | 0.06 (0.02-0.19)^b^ |
|  | **Financial hardship** | | | | | | |
|  |  | No (reference) | N/A | N/A | N/A | N/A | 1.00 |
|  |  | Yes | N/A | N/A | N/A | N/A | 2.81 (1.72-4.6)^b^ |
|  | **Remoteness** | | | | | | |
|  |  | Major cities (reference) | N/A | N/A | N/A | 1.00 | 1.00 |
|  |  | Inner regional | N/A | N/A | N/A | 1.23 (0.83-1.82) | 0.86 (0.57-1.31) |
|  |  | Outer regional | N/A | N/A | N/A | 3.23 (2.07-5.04)^b^ | 2.37 (1.47-3.82)^b^ |
|  |  | Remote/very remote | N/A | N/A | N/A | 2.48 (0.86-7.11) | 2.05 (0.61-6.84) |
| **Relative outcome (internet access: no, other reasons)** | | | | | | | |
|  | **Mental health problems** | | | | | | |
|  |  | No (reference) | 1.00 | 1.00 | 1.00 | 1.00 | 1.00 |
|  |  | Yes | 1.33 (1.06-1.67)^c^ | 1.61 (1.26-2.05)^b^ | 1.38 (1.07-1.78)^c^ | 1.39 (1.08-1.80)^c^ | 1.11 (0.85-1.44) |
|  | **Sex** | | | | | | |
|  |  | Male (reference) | N/A | 1.00 | 1.00 | 1.00 | 1.00 |
|  |  | Female | N/A | 1.07 (0.91-1.25) | 0.92 (0.78-1.08) | 0.93 (0.79-1.10) | 0.85 (0.71-1.01) |
|  | **Age group (years)** | | | | | | |
|  |  | <35 (reference) | N/A | 1.00 | 1.00 | 1.00 | 1.00 |
|  |  | 35-54 | N/A | 1.84 (1.30-2.59)^e^ | 3.43 (2.40-4.90)^b^ | 3.29 (2.30-4.70)^b^ | 2.82 (1.92-4.12)^b^ |
|  |  | >54 | N/A | 11.03 (8.29-14.67)^b^ | 15.64 (11.66-20.98)^b^ | 14.55 (10.83-19.56)^b^ | 6.54 (4.71-9.07)^b^ |
|  | **Partner status** | | | | | | |
|  |  | No partner (reference) | N/A | N/A | 1.00 | 1.00 | 1.00 |
|  |  | Have a partner | N/A | N/A | 0.25 (0.21-0.30)^b^ | 0.25 (0.21-0.29)^b^ | 0.37 (0.31-0.44)^b^ |
|  | **Children aged <15 years** | | | | | | |
|  |  | No (reference) | N/A | N/A | 1.00 | 1.00 | 1.00 |
|  |  | Yes | N/A | N/A | 0.45 (0.33-0.60)^b^ | 0.45 (0.34-0.61)^b^ | 0.56 (0.41-0.77)^b^ |
|  | **Employment status** | | | | | | |
|  |  | Employed (reference) | N/A | N/A | N/A | N/A | 1.00 |
|  |  | Unemployed | N/A | N/A | N/A | N/A | 1.63 (0.90-2.96) |
|  |  | Not in the labor force | N/A | N/A | N/A | N/A | 1.92 (1.56-2.35)^b^ |
|  | **Annual household gross income** | | | | | | |
|  |  | <AU $34,000 (US $ 21,715) (reference) | N/A | N/A | N/A | N/A | 1.00 |
|  |  | AU $34,000-AU $59,999 (US $21,715-US $38,320) | N/A | N/A | N/A | N/A | 0.70 (0.56-0.86)^e^ |
|  |  | AU $60,000-AU $99,999 (US $ 38,321-US $63,867) | N/A | N/A | N/A | N/A | 0.34 (0.26-0.44)^b^ |
|  |  | AU $100,000-AU $159,999 (US $63,868-US $ 102,187) | N/A | N/A | N/A | N/A | 0.20 (0.14-0.27)^b^ |
|  |  | >AU $160,000 (US $102,188) | N/A | N/A | N/A | N/A | 0.12 (0.07-0.19)^b^ |
|  | **Financial hardship** | | | | | | |
|  |  | No (reference) | N/A | N/A | N/A | N/A | 1.00 |
|  |  | Yes | N/A | N/A | N/A | N/A | 1.23 (0.99-1.54) |
|  | **Remoteness** | | | | | | |
|  |  | Major cities (reference) | N/A | N/A | N/A | 1.00 | 1.00 |
|  |  | Inner regional | N/A | N/A | N/A | 1.92 (1.60-2.30)^b^ | 1.49 (1.23-1.82)^b^ |
|  |  | Outer regional | N/A | N/A | N/A | 2.31 (1.84-2.91)^b^ | 1.74 (1.37-2.21)^b^ |
|  |  | Remote/very remote | N/A | N/A | N/A | 2.38 (1.41-4.02)^e^ | 2.32 (1.38-3.90)^e^ |

^a^RRR: relative risk ratio.

^b^*P*<.001.

^c^*P*<.05.

^d^N/A: not applicable.

^e^*P*<.01.
