# Supplementary material for: Mental Health Problems and Internet Access: Results From an Australian National Household Survey
Source: JMIR Ment Health. 2020 May 15;7(5):e14825. doi: 10.2196/14825 (PMC7260658; doi:10.2196/14825)
Supplement: Multimedia Appendix 2 [file mental_v7i5e14825_app2.docx]

| Base outcome (internet access: yes) | | | Model 1, RRR^a^ (95% CI) | Model 2 (adding sex and age), RRR (95% CI) | Model 3 (adding partner status and children aged <15 years ), RRR (95% CI) | Model 4 (adding remoteness), RRR (95% CI) | Model 5 (adding employment, household income, and financial hardship), RRR (95% CI) |
| --- | --- | --- | --- | --- | --- | --- | --- |
| **Relative outcome (internet access: no, cannot afford)** | | | | | | | |
|  | **Severe mental health conditions** | | | | | | |
|  |  | No (reference) | 1.00 | 1.00 | 1.00 | 1.00 | 1.00 |
|  |  | Yes | 4.15 (2.68-6.42)^b^ | 4.29 (2.77-6.62)^b^ | 3.38 (2.16-5.29)^b^ | 3.46 (2.20-5.45)^b^ | 1.92 (1.16-3.19)^c^ |
|  | **Sex** | | | | | | |
|  |  | Male (reference) | N/A^d^ | 1.00 | 1.00 | 1.00 | 1.00 |
|  |  | Female | N/A | 1.30 (0.89-1.90) | 1.17 (0.77-1.76) | 1.17 (0.78-1.77) | 0.94 (0.58-1.51) |
|  | **Age group (years)** | | | | | | |
|  |  | <35 (reference) | N/A | 1.00 | 1.00 | 1.00 | 1.00 |
|  |  | 35-54 | N/A | 0.63 (0.39-1.02) | 1.21 (0.73-2.02) | 1.19 (0.72-1.98) | 0.87 (0.51-1.51) |
|  |  | >54 | N/A | 1.15 (0.72-1.82) | 2.61 (1.47-4.64)^e^ | 2.46 (1.39-4.37)^e^ | 1.71 (0.86-3.40) |
|  | **Partner status** | | | | | | |
|  |  | No partner (reference) | N/A | N/A | 1.00 | 1.00 | 1.00 |
|  |  | Have a partner | N/A | N/A | 0.15 (0.10-0.21)^b^ | 0.14 (0.10-0.21)^b^ | 0.22 (0.15-0.33)^b^ |
|  | **Children aged <15 years** | | | | | | |
|  |  | No (reference) | N/A | N/A | 1.00 | 1.00 | 1.00 |
|  |  | Yes | N/A | N/A | 1.97 (1.23-3.14)^e^ | 1.90 (1.19-3.04)^e^ | 2.08 (1.19-3.62)^c^ |
|  | **Employment status** | | | | | | |
|  |  | Employed (reference) | N/A | N/A | N/A | N/A | 1.00 |
|  |  | Unemployed | N/A | N/A | N/A | N/A | 1.96 (0.96-4.01) |
|  |  | Not in the labor force | N/A | N/A | N/A | N/A | 1.28 (0.76-2.17) |
|  | **Annual household gross income** | | | | | | |
|  |  | <AU $34,000 (US $ 21,715) (reference) | N/A | N/A | N/A | N/A | 1.00 |
|  |  | AU $34,000-AU $59,999 (US $21,715-US $38,320) | N/A | N/A | N/A | N/A | 0.99 (0.58-1.69) |
|  |  | AU $60,000-AU $99,999 (US $ 38,321-US $63,867) | N/A | N/A | N/A | N/A | 0.45 (0.26-0.77)^e^ |
|  |  | AU $100,000-AU $159,999 (US $63,868-US $ 102,187) | N/A | N/A | N/A | N/A | 0.06 (0.03-0.15)^b^ |
|  |  | >AU $160,000 (US $102,188) | N/A | N/A | N/A | N/A | 0.06 (0.02-0.19)^b^ |
|  | **Financial hardship** | | | | | | |
|  |  | No (reference) | N/A | N/A | N/A | N/A | 1.00 |
|  |  | Yes | N/A | N/A | N/A | N/A | 2.94 (1.78-4.88)^b^ |
|  | **Remoteness** | | | | | | |
|  |  | Major cities (reference) | N/A | N/A | N/A | 1.00 | 1.00 |
|  |  | Inner regional | N/A | N/A | N/A | 1.25 (0.85-1.85) | 0.89 (0.59-1.35) |
|  |  | Outer regional | N/A | N/A | N/A | 3.28 (2.10-5.13)^b^ | 2.43 (1.50-3.93)^b^ |
|  |  | Remote/very remote | N/A | N/A | N/A | 2.72 (1.05-7.02)^c^ | 2.02 (0.61-6.70) |
| **Relative outcome (internet access: no, other reasons)** | | | | | | | |
|  | **Severe mental health conditions** | | | | | | |
|  |  | No (reference) | 1.00 | 1.00 | 1.00 | 1.00 | 1.00 |
|  |  | Yes | 2.41 (1.80-3.23)^b^ | 2.52 (1.80-3.52)^b^ | 2.03 (1.45-2.84)^b^ | 2.06 (1.46-2.91)^b^ | 1.43 (1.01-2.01)^c^ |
|  | **Sex** | | | | | | |
|  |  | Male (reference) | N/A | 1.00 | 1.00 | 1.00 | 1.00 |
|  |  | Female | N/A | 1.07 (0.91-1.25) | 0.92 (0.78-1.08) | 0.93 (0.79-1.09) | 0.85 (0.71-1.01) |
|  | **Age group (years)** | | | | | | |
|  |  | <35 (reference) | N/A | 1.00 | 1.00 | 1.00 | 1.00 |
|  |  | 35-54 | N/A | 1.80 (1.27-2.53)^e^ | 3.30 (2.31-4.73)^b^ | 3.17 (2.22-4.54)^b^ | 2.76 (1.88-4.04)^b^ |
|  |  | >54 | N/A | 10.86 (8.16-14.46)^b^ | 15.55 (11.59-20.87)^b^ | 14.47 (10.76-19.46)^b^ | 6.66 (4.81-9.23)^b^ |
|  | **Partner status** | | | | | | |
|  |  | No partner (reference) | N/A | N/A | 1.00 | 1.00 | 1.00 |
|  |  | Have a partner | N/A | N/A | 0.26 (0.22-0.30)^b^ | 0.25 (0.21-0.29)^b^ | 0.37 (0.31-0.45)^b^ |
|  | **Children aged <15 years** | | | | | | |
|  |  | No (reference) | N/A | N/A | 1.00 | 1.00 | 1.00 |
|  |  | Yes | N/A | N/A | 0.46 (0.34-0.61)^b^ | 0.46 (0.35-0.62)^b^ | 0.57 (0.42-0.78)^e^ |
|  | **Employment status** | | | | | | |
|  |  | Employed (reference) | N/A | N/A | N/A | N/A | 1.00 |
|  |  | Unemployed | N/A | N/A | N/A | N/A | 1.59 (0.89-2.86) |
|  |  | Not in the labor force | N/A | N/A | N/A | N/A | 1.86 (1.52-2.29)^b^ |
|  | **Annual household gross income** | | | | | | |
|  |  | <AU $34,000 (US $ 21,715) (reference) | N/A | N/A | N/A | N/A | 1.00 |
|  |  | AU $34,000-AU $59,999 (US $21,715-US $38,320) | N/A | N/A | N/A | N/A | 0.68 (0.55-0.84)^b^ |
|  |  | AU $60,000-AU $99,999 (US $ 38,321-US $63,867) | N/A | N/A | N/A | N/A | 0.33 (0.26-0.43)^b^ |
|  |  | AU $100,000-AU $159,999 (US $63,868-US $ 102,187) | N/A | N/A | N/A | N/A | 0.19 (0.14-0.27)^b^ |
|  |  | >AU $160,000 (US $102,188) | N/A | N/A | N/A | N/A | 0.12 (0.07-0.19)^b^ |
|  | **Financial hardship** | | | | | | |
|  |  | No (reference) | N/A | N/A | N/A | N/A | 1.00 |
|  |  | Yes | N/A | N/A | N/A | N/A | 1.22 (0.99-1.52) |
|  | **Remoteness** | | | | | | |
|  |  | Major cities (reference) | N/A | N/A | N/A | 1.00 | 1.00 |
|  |  | Inner regional | N/A | N/A | N/A | 1.91 (1.59-2.30)^b^ | 1.49 (1.23-1.81)^b^ |
|  |  | Outer regional | N/A | N/A | N/A | 2.32 (1.84-2.92)^b^ | 1.74 (1.37-2.21)^b^ |
|  |  | Remote/very remote | N/A | N/A | N/A | 2.38 (1.41-4.01)^e^ | 2.35 (1.40-3.93)^e^ |

^a^RRR: relative risk ratio.

^b^*P*<.001.

^c^*P*<.05.

^d^N/A: not applicable.

^e^*P*<.01.
