# Supplementary material for: Mental Health Problems and Internet Access: Results From an Australian National Household Survey
Source: JMIR Ment Health. 2020 May 15;7(5):e14825. doi: 10.2196/14825 (PMC7260658; doi:10.2196/14825)
Supplement: Multimedia Appendix 3 [file mental_v7i5e14825_app3.docx]

|  | Psychological distress | | | |
| --- | --- | --- | --- | --- |
|  | Lower | | Very high | |
|  | n (%) | 95% CI | n (%) | 95% CI |
| **Persons with internet access at home** | | | | |
| Yes | 13152 (91.94) | 91.40‒92.44 | 861 (87.42) | 84.56‒89.81 |
| No | 1247 (8.06) | 7.56‒8.60 | 139 (12.58) | 10.19‒15.44 |
