# Supplementary material for: Mental Health Problems and Internet Access: Results From an Australian National Household Survey
Source: JMIR Ment Health. 2020 May 15;7(5):e14825. doi: 10.2196/14825 (PMC7260658; doi:10.2196/14825)
Supplement: Multimedia Appendix 4 [file mental_v7i5e14825_app4.docx]

|  | Model 1  OR^a^ (95% CI) | Model 2  (adding sex and age)  OR (95% CI) | Model 3  (adding partner status and children under 15 years old)  OR (95% CI) | Model 4  (adding remoteness)  OR (95% CI) | Model 5  (adding employment, household income and financial hardship)  OR (95% CI) |
| --- | --- | --- | --- | --- | --- |
| **Psychological distress** |  |  |  |  |  |
| Lower (reference) | 1.00 | 1.00 | 1.00 | 1.00 | 1.00 |
| Very high | 1.64 (1.28‒2.10)^b^ | 2.30 (1.77‒2.98)^b^ | 2.08 (1.58‒2.75)^b^ | 2.14 (1.61‒2.84)^b^ | 1.46 (1.08−1.97)^c^ |
| **Sex** |  |  |  |  |  |
| Male (reference) | N/A | 1.00 | 1.00 | 1.00 | 1.00 |
| Female | N/A | 1.15 (1.00‒1.32) | 1.00 (0.87‒1.16) | 1.01 (0.87‒1.17) | 0.92 (0.79−1.07) |
| **Age group** |  |  |  |  |  |
| < 35 (reference) | N/A | 1.00 | 1.00 | 1.00 | 1.00 |
| 35‒54 | N/A | 1.42 (1.09‒1.85)^c^ | 2.49 (1.89‒3.27) ^b^ | 2.40 (1.82‒3.15)^b^ | 1.99 (1.49−2.65)^b^ |
| > 54 | N/A | 8.72 (6.99‒10.89)^b^ | 12.92 (10.21‒16.36)^b^ | 11.96 (9.42‒15.19)^b^ | 4.92 (3.79−6.39)^b^ |
| **Partner status** |  |  |  |  |  |
| No partner (reference) | N/A | N/A | 1.00 | 1.00 | 1.00 |
| Have a partner | N/A | N/A | 0.26 (0.22‒0.30)^b^ | 0.25 (0.22‒0.30)^b^ | 0.39 (0.33−0.46)^b^ |
| **Children under 15 years old** |  |  |  |  |  |
| No (reference) | N/A | N/A | 1.00 | 1.00 | 1.00 |
| Yes | N/A | N/A | 0.67 (0.51‒0.87)^e^ | 0.66 (0.51‒0.86)^e^ | 0.79 (0.60−1.05) |
| **Employment status** |  |  |  |  |  |
| Employed (reference) | N/A | N/A | N/A | N/A | 1.00 |
| Unemployed | N/A | N/A | N/A | N/A | 0.98 (0.60−1.61) |
| Not in the labour force | N/A | N/A | N/A | N/A | 2.40 (2.01−2.87)^b^ |
| **Annual household gross income** |  |  |  |  |  |
| <AU $34,000 (US $ 21,715) (reference) | N/A | N/A | N/A | N/A | 1.00 |
| AU $34,000-AU $59,999 (US $21,715-US $38,320) | N/A | N/A | N/A | N/A | 0.63 (0.52−0.75)^b^ |
| AU $60,000-AU $99,999 (US $ 38,321-US $63,867) | N/A | N/A | N/A | N/A | 0.37 (0.30−0.47)^b^ |
| AU $100,000-AU $159,999 (US $63,868-US $ 102,187) | N/A | N/A | N/A | N/A | 0.18 (0.13−0.25)^b^ |
| >AU $160,000 (US $102,188) | N/A | N/A | N/A | N/A | 0.12 (0.08−0.17)^b^ |
| **Financial hardship** |  |  |  |  |  |
| No (reference) | N/A | N/A | N/A | N/A | 1.00 |
| Yes | N/A | N/A | N/A | N/A | 1.22 (1.00−1.48) |
| **Remoteness** |  |  |  |  |  |
| Major cities (reference) | N/A | N/A | N/A | 1.00 | 1.00 |
| Inner regional | N/A | N/A | N/A | 1.81 (1.54‒2.13)^b^ | 1.42 (1.20−1.69)^b^ |
| Outer regional | N/A | N/A | N/A | 2.68 (2.15‒3.34)^b^ | 2.12 (1.68−2.68)^b^ |
| Remote/very remote | N/A | N/A | N/A | 3.17 (1.93‒5.19)^b^ | 3.87 (2.21−6.79)^b^ |

^a^p< 0.001; ^b^p<0.01; ^c^p<0.05.

^a^OR: odd ratio.

^b^*P*<.001.

^c^*P*<.05.

^d^N/A: not applicable.

^e^*P*<.01.
